# Primate occurrence across a human-impacted landscape in Guinea-Bissau and neighbouring regions in West Africa: using a systematic literature review to highlight the next conservation steps

**DOI:** 10.7717/peerj.4847

**Published:** 2018-05-23

**Authors:** Elena Bersacola, Joana Bessa, Amélia Frazão-Moreira, Dora Biro, Cláudia Sousa, Kimberley Jane Hockings

**Affiliations:** 1 Centre for Research in Anthropology (CRIA/NOVA FCSH), Lisbon, Portugal; 2 Anthropological Centre for Conservation, the Environment and Development (ACCEND), Department of Humanities and Social Sciences, Oxford Brookes University, Oxford, United Kingdom; 3 Department of Zoology, University of Oxford, Oxford, United Kingdom; 4 Department of Anthropology, Faculty of Social Sciences and Humanities, Universidade NOVA de Lisboa, Lisbon, Portugal; 5 Centre for Ecology and Conservation, College of Life and Environmental Sciences, University of Exeter, Cornwall, United Kingdom

**Keywords:** King colobus, Endangered species, West Africa, Red colobus, Chimpanzee, Primate conservation, Wildlife surveys, Guinea-Bissau, Senegal, Guinea

## Abstract

**Background:**

West African landscapes are largely characterised by complex agroforest mosaics. Although the West African forests are considered a nonhuman primate hotspot, knowledge on the distribution of many species is often lacking and out-of-date. Considering the fast-changing nature of the landscapes in this region, up-to-date information on primate occurrence is urgently needed, particularly of taxa such as colobines, which may be more sensitive to habitat modification than others. Understanding wildlife occurrence and mechanisms of persistence in these human-dominated landscapes is fundamental for developing effective conservation strategies.

**Methods:**

In this paper, we aim to review current knowledge on the distribution of three threatened primates in Guinea-Bissau and neighbouring regions, highlighting research gaps and identifying priority research and conservation action. We conducted a systematic literature review of primate studies from 1976 to 2016 in Guinea-Bissau, southern Senegal and western Guinea (Boké Region). We mapped historical observation records of chimpanzee (*Pan troglodytes verus*), Temminck’s red colobus (*Pilicolobus badius temminckii*) and king colobus (*Colobus polykomos*), including our preliminary survey data from Dulombi, a newly established National Park (NP) in Guinea-Bissau.

**Results:**

We found 151 documents, including 87 journal articles, that contained field data on primates in this region. In Guinea-Bissau, nearly all studies focussed south of the Corubal River, including mainly Cantanhez, Cufada, and Boé NP’s. In Senegal, most of the data came from Fongoli and Niokolo-Koba NP. In Boké (Guinea) studies are few, with the most recent data coming from Sangarédi. In Dulombi NP we recorded eight primate species, including chimpanzees, red colobus and king colobus*.* Across the selected region, chimpanzees, red colobus and king colobus were reported in eleven, twelve and seven protected areas, respectively.

**Discussion:**

Our study demonstrates large geographical research gaps particularly for the two colobines. For the first time after more than two decades, we confirm the presence of red colobus and king colobus north of the Corubal River in Guinea-Bissau. The little information available from large parts of the red colobus range raises questions regarding levels of population fragmentation in this species, particularly in Casamance and across northern Guinea-Bissau. There are still no records demonstrating the occurrence of king colobus in Senegal, and the presence of a viable population in north-eastern Guinea-Bissau remains uncertain. While the occurrence of chimpanzees in Guinea-Bissau and Senegal is well documented, data from Boké (Guinea) are sparse and out-of-date. Our approach—the mapping of data gathered from a systematic literature review—allows us to provide recommendations for selecting future geographical survey locations and planning further research and conservation strategies in this region.

## Introduction

The present-day West African natural landscape is largely characterised by mosaics of different forest types, agriculture, road infrastructures and human settlements, rather than continuous intact forest blocks (Norris et al., 2010). Although rich in biodiversity, West Africa has one of the fastest growing human populations. Spatially-explicit land-change models suggest that by 2030 West Africa will have experienced one of the world’s highest rates of urban development, further increasing the demand for land and natural resources (Seto, Güneralp & Hutyra, 2012). While the human population growth is mainly concentrated in urban areas, in several countries the number of people living in rural settings is also quickly increasing. For example, in 2016 the rural population in the Republic of Guinea was double that of 1986 (World Bank, 2018a). Consequently, conservation strategies that include the management of key biodiversity areas, alongside working closely with local human populations, are urgently required in this region.

The human-dominated characteristics of the landscape in West Africa make designating large, unmodified protected areas in land use plans often not possible. For example, 1% and none of the forest cover in Guinea and Guinea-Bissau can be classified as primary, respectively (FAO, 2015). Therefore, alternative approaches that recognise the value of agro-forest landscapes for biodiversity conservation may be more suitable. For similar reasons, in Europe only 27% of the protected landscape is managed under the IUCN Categories I–II, i.e., large, mostly untouched areas where management focuses on strict protection, 41% of which is in Sweden and Norway (EEA, 2017). The majority (58%) of protected territory in Europe falls under Categories V–VI, which employ the sustainable use of natural resources inside human-dominated protected areas as a measure to achieve ecosystem/biodiversity conservation (IUCN, 2016).

Effective conservation management strategies need to be based on data from the biological and social sciences. For example, when developing land use plans, data on species distribution patterns and ecological requirements are fundamental, because these allow conservationists to select zones that include the space and resources required for the species’ long term persistence (Margules & Pressey, 2000). Conservation threats and patterns of human-wildlife coexistence need to be identified at the regional and local level, and their mechanisms must be understood from both biological and social perspectives (Humle & Hill, 2016). Additionally, integrating sociocultural and political aspects, and involving local people in conservation policies, will help reduce conservation conflicts (Andrade & Rhodes, 2012).

West Africa is a nonhuman primate (hereafter primate) hotspot (Oates, 2011), but data are lacking on the presence and distribution of primates in many parts of this region (Gardner et al., 2009). For gathering this information, systematic methods for estimating primate population densities (e.g., distance sampling: Buckland et al., 2015), and investigating distribution patterns (e.g., occupancy models: MacKenzie, 2006) are well-established. Although these methods can offer robust quantitative data and are easily replicated (e.g., for monitoring programs), reconnaissance surveys are necessary as the first step to gather crucial information on species presence and environmental characteristics (Campbell et al., 2016). This first inspection helps researchers to select areas for future systematic approaches that require more time and/or financial efforts.

Acknowledging the lack of data on primate distributions, for this study we aimed to identify the crucial next steps of primate conservation research and strategies in Guinea-Bissau and the neighbouring areas (southern Senegal and Boké region in Guinea), by: (1) conducting a systematic literature review of primate studies carried out in the region, (2) presenting data from primate reconnaissance surveys at Dulombi NP, and (3) mapping the distribution of the most threatened primates (chimpanzees, red colobus and king colobus) in the region based on our survey and historical records. Our ultimate goal is to provide useful guidelines for scientists and conservation practitioners when developing future research and conservation management strategies in this region.

## Study Area

Guinea-Bissau is a small country (36,125 km^2^) located within the Guinean forest-savannah mosaic ecoregion, which separates the Guinean moist forests in the south and the West Sudanian savanna in the north (Olson et al., 2001). The climate in Guinea-Bissau is characterised by a hot wet season from June to October and a hot dry season from November to May. The average monthly rainfall during the wet season is 298.2 mm (World Bank, 2018b), whereas the rest of the year is distinguished by an almost complete absence of rain. The dry season also marks the lowest and highest annual temperatures, specifically in December-January (25 °C) and May (29 °C), respectively. Guinea-Bissau is included in the Casamance regional community of African primates, one of the eleven distinct communities recognised by the IUCN/SSC Primate Specialist Group (Oates, 1996), characterised by two distinct species: Guinea baboon (*Papio papio*) and Temminck’s red colobus (*Piliocolobus badius temminckii*). The southern part of the country includes the forest-savannah belt, with more humid forests covering the south and drier savannah-riparian forest mosaics in the east. Guinea-Bissau contains 34 PAs, which account for 16% and 10% of the terrestrial and marine national territory, respectively (UNEP-WCMC & IUCN, 2018). The majority of PAs in Guinea-Bissau are managed by the Institute for Biodiversity and Protected Areas (IBAP). Several globally significant primate species are present in the region, including the Critically Endangered West African chimpanzee (*Pan troglodytes verus*), the Temminck’s red colobus and the king colobus (*Colobus polykomos*) (Limoges, 1989; IUCN, 2018). The conservation status of the two colobines was reassessed in 2016 at the IUCN SSC African primate Red List Assessment workshop in Rome. According to the Red List Authority Focal Point Dr. Christoph Schwitzer, during the reassessment “the Temminck’s red colobus was uplisted from Endangered to Critically Endangered, and the king colobus was uplisted from Vulnerable to Endangered. The draft assessments are in the process of being written up and will be subject to peer-review before the new categories are confirmed. They are likely to go live on the Red List website towards the end of 2018” (C Schwitzer, pers. comm., 2018). Guinea-Bissau represents the westernmost limit for chimpanzees and a significant portion of the red colobus geographical range. Previous population estimates for chimpanzees in Guinea-Bissau range from 137 individuals in Lagoas de Cufada Natural Park (Carvalho, Marques & Vicente, 2013) to approximately 700 individuals in the Boé region (Serra, Silva & Lopes, 2007). Within Cantanhez NP previous estimates ranged between 376 and 2,632 individuals (Torres et al., 2010), with a density of 1.1-6.18 weaned individuals/km^2^ within three key forests in central Cantanhez (Sousa et al., 2011). Estimates of the effective population sizes (i.e., the number of individuals in a population that contribute to the next reproductive generation) for red colobus and king colobus in Cantanhez exist (Minhós et al., 2016), but no population density estimates from censuses are available for either species.

In 1982 the Dulombi area was identified as a biodiversity hotspot (Chardonnet, 1983), and guidelines were developed for a wildlife management project. Dulombi was subsequently proposed as a Forest Reserve/National Park in the early 1990s (Paris, 1991; Thibault, 1993), but was never officially gazetted. Since 2000 the Appui à la Gestion Intégrée des Ressources (AGIR) and IBAP developed a plan for establishing a network of Protected Areas in the southeast, including two new National Parks (Dulombi and Boé), as well as three connecting corridors (Salifo, Cuntabane, Tchetché). The goal of this new PA network, collectively known as the Dulombi-Boé-Tchetché Complex (DBT), was to increase terrestrial biodiversity conservation efforts in the southeast, and establish a trans-frontier PA network with the neighbouring Republic of Guinea (a project known as APT-B1) (UNDP, 2009). In 2002 the Action Plan for the West African chimpanzee recognised the Fouta Djallon region of Guinea and Guinea-Bissau as a chimpanzee priority area for conservation (Kormos et al., 2003). Although data are scarce, the DBT also represents one of the last refuges for lions (*Panthera leo*), leopards (*Panthera pardus*) and African elephants (*Loxodonta africana*) in the western part of their range (Brugiere et al., 2005; Brugière et al., 2006). The first floristic inventory in the DBT classified the vegetation into three main types: forests, savannah-grasslands and agriculture (Catarino & Palminha, 2014). Forest habitats include riparian forest, open forest, savannah-woodland and palm strands. Riparian forest and palm strands are normally present along streams and seasonally flooded areas at lower altitudes, whereas open forest and savannah-woodland occur at slightly higher altitudes, often acting as a buffer zone between riparian forest and grasslands. Similarly to the majority of protected areas in Guinea-Bissau (e.g., Cantanhez NP, (Hockings & Sousa, 2013), people are present inside the park and mainly practice subsistence agriculture (including shifting agriculture and cashew orchards) and hunt for bushmeat (including monkeys, medium and large-sized ungulates and rodents). The majority of people belong to the Fula ethnic group, but Balanta and other ethnicities are also present. During the cashew season (March–July), people from neighbouring Guinea arrive in the park in search of work in the orchards.

## Methods

In this paper we combined a systematic literature review of primate studies conducted in Guinea-Bissau and neighbouring areas with our survey data from Dulombi NP, Guinea-Bissau, to establish knowledge gaps on primate distributions in this region. All research involving wild primates is non-invasive and complied with the ethics guidelines detailed by the Association for the Study of Animal Behaviour (UK) and to the legal requirements of Guinea-Bissau in which the research was conducted. The research was conducted on protected species in Dulombi National Park and was approved by the Institute for Biodiversity and Protected Areas (IBAP), Guinea-Bissau.

### Literature search strategy

**Figure 1:**
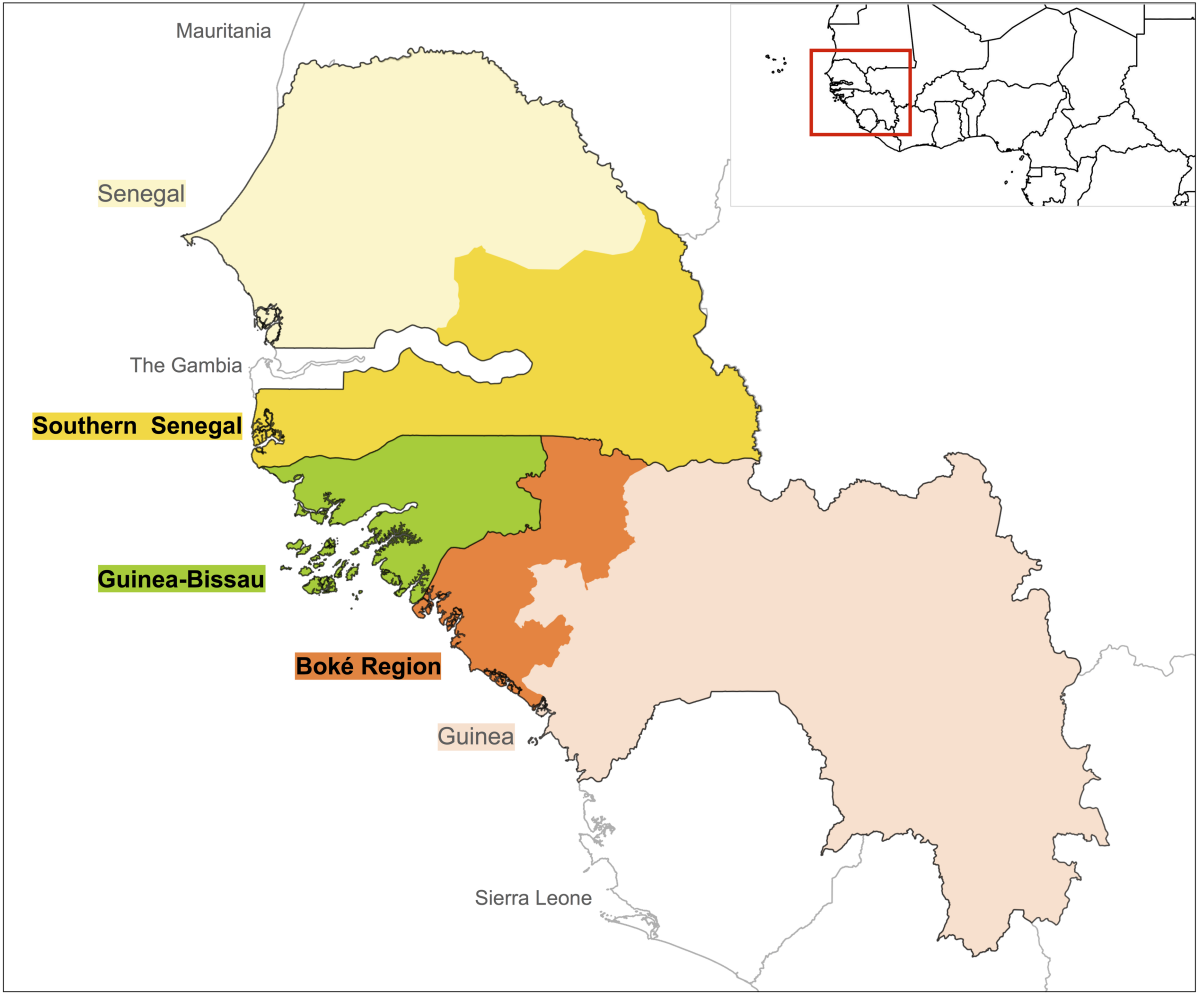
Geographical areas included in the literature search: Guinea-Bissau (green), Southern Senegal (yellow) and Boké Region in Guinea (orange).

We carried out a systematic literature review of field-based primatological studies conducted in the past 40 years (1976–2016) in Guinea-Bissau, and the bordering regions of southern Senegal and northwest Guinea. We included published and unpublished documents that contained data collected directly from primate surveys, behavioural and ecological studies, as well as information gathered using indirect methods such as interviews with local people. EB and KJH searched for published material using Web of Science and Google Scholar using the following keywords: ‘chimpanzee’, ‘colobus’, ‘baboon’, ‘*temminckii*’, ‘*polykomos*’, ‘*sabaeus*’, ‘patas’, ‘primate survey’, ‘Guinea’, ‘Guinea-Bissau’, ‘Senegal’. The following is an example of a full search in Web of Science: (chimpanzee* Guinea OR chimpanzee* Guinea-Bissau OR chimpanzee* Senegal OR colobus* Guinea OR colobus* Guinea-Bissau OR colobus* Senegal OR primate surveys Guinea OR primate surveys Guinea-Bissau OR primate surveys Senegal). In Senegal, we selected studies conducted in the regions bordering Guinea-Bissau and Guinea, i.e., Ziguinchor, Sédhiou, Kolda, Tambacounda and Kédougou. For Guinea we only selected studies conducted in the Boké Region (Fig. 1). We included published articles, technical reports, PhD and Masters dissertations, books, book chapters and meeting abstracts. For the grey literature we reviewed our collections, searched citations from published articles and searched websites of organisations working in the region (e.g., Wild Chimpanzee Foundation, IBAP, Chimbo). When data from Masters dissertations were published we only included published documents. We also included unpublished data presented from other sources such as reviews and status assessments. Discrepancies between reviewers were resolved through discussions, and when necessary, with a third contributor (JB).

### Primate surveys in Dulombi National Park

**Figure 2:**
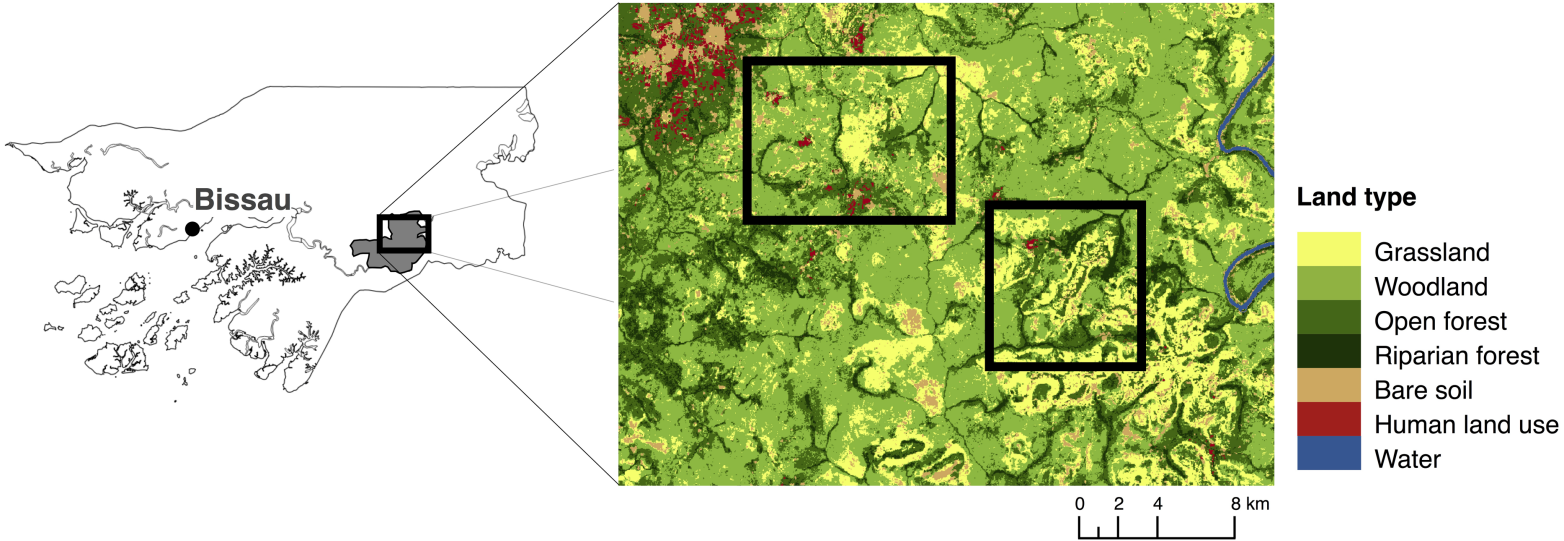
Map and location of study area. Dulombi National Park is highlighted in grey.

We (EB and JB) carried out surveys in October and November 2015 in two areas in Dulombi NP, one around Dulombi village (N11°51.62–W14°30.17) and one South and East of Paiai Lumba village (N11°50.20–W14°25.23, Fig. 2). To gather preliminary information on primate occurrence, we collected data using reconnaissance surveys (recces) and camera traps (Rovero et al., 2013; Campbell et al., 2016). We walked 16 recces for a total of 92 km on human-made and animal trails and on least-resistance parts of the habitats across savannah-grassland, savannah-woodland, open forest, riparian forest and agricultural patches. Due to reports of chimpanzees ranging east of Paiai Lumba, we chose to maximise the survey efforts in this area. We walked 16 km around Dulombi and 76 km around Paiai Lumba villages. We travelled at an average walking speed of 1–1.5 km/hour. Visibility on each side varied from c. 20 m within forest to c. 100 m in savannah habitats. With each animal detection we recorded species, time, group composition, response behaviour, habitat type and GPS coordinates. Where we heard primate vocalisations but could not see the individual(s), we took compass bearings and estimated distances to the nearest 50 m. We later examined GoogleEarth maps to establish the approximate locations of the vocalisations and, when evident, the type of habitat. Individuals of the same species were considered from the same group if sighted within 100 m distance from the previous ones. We recorded and identified all indirect signs of primate presence, such as footprints and feeding traces, as well as nests and dung remains (for chimpanzees), with the help of experienced local guides. Chimpanzee faeces and feeding traces are clearly distinguishable from those of other primates due to the size, form, type of food and patterns of consumption (Bessa, Sousa & Hockings, 2015). If a trace was ambiguous it was not attributed to a particular species. We counted chimpanzee nests and recorded tree species, the number of nests within the same cluster, and took a GPS point. We calculated the encounter rate for each primate detected during recce walks (heard and/or sighted) from the number of groups detected divided by km walked.

We deployed 21 camera traps (Bushnell Trophy Cam Aggressor HD Low-Glow 119774, heat-motion trigger) across forest habitats (twelve in riparian forest, two in open forest, and four in woodland) and agricultural fields (one in rice *Oryza sp.* and two in peanut *Arachis hypogaea* farms near Dulombi). The cameras were set up to record primate behaviour (video/hybrid mode, 10s interval), and did not follow a systematic spatial survey design. For example, camera points were not independent because some cameras were set up 50–100 m from one another. We therefore did not attempt to provide indices of abundance from the camera traps but simply indicated which species of primates were detected and in what habitat.

### Mapping primate occurrence

We mapped study locations and all presence records of chimpanzee, red colobus and king colobus from published and unpublished studies using a grid system where each square cell measured 25 km^2^. We chose to use 5 × 5 km cells because several studies provided names of localities rather than GPS coordinates of observations. We reviewed each source and selected the grid cells that overlapped with the study area and where primates were reported present. Where exact coordinates were not reported we mapped the approximate location using the name of the village and the map presented in the study. Because the geographical areas of research carried out between 1976 and 1985 overlapped with research conducted in later years, here we present maps showing studies conducted since 1986. Mapping was performed in QGIS v. 2.14.

## Results

### Literature search

**Figure 3:**
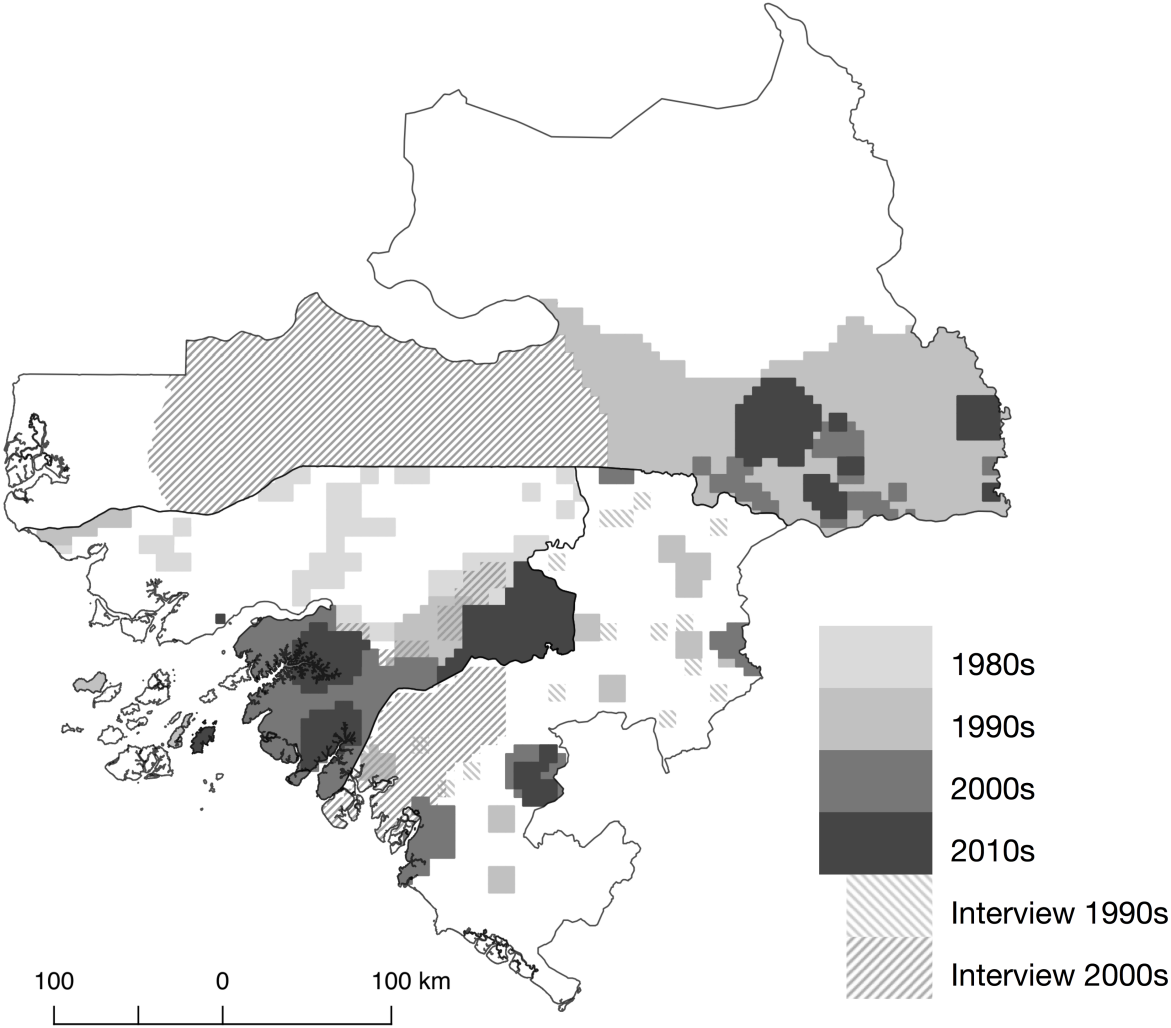
Map and location of field-based research carried out that includes data on primates in Guinea-Bissau and neighbouring regions.

We found a total of 151 documents that focused on or included data on primates in the selected region since 1976 (the last search was conducted in September 2016). These included 87 published journal articles, 16 technical reports, five book chapter, nine PhD theses, two Masters dissertations, two books and 30 meeting abstracts. Fifty-four were from Guinea-Bissau, 14 from the Boké Region in Guinea, 78 from southern Senegal, and five documents included data from two or all three regions. The majority of journal articles and technical reports (*N* = 98) contained data from surveys and observations, including direct sightings, camera trap data and/or inferences from signs/traces/biological samples. Fourteen journal articles included information collected using interviews. Within Guinea-Bissau, research on primates mainly focused on areas south of the Corubal River (*N* = 47, Fig. 3), coinciding with the region with highest forest cover (Tombali) and where most of the chimpanzee population in Guinea-Bissau occur (Boé, Tombali and Quinara regions). Only one site in Guinea-Bissau at Caiquene-Cadique in Cantanhez NP (Tombali region) has ongoing research efforts to monitor and collect behavioural data on a specific chimpanzee community (Hockings & Sousa, 2012; Hockings & Sousa, 2013; Bessa, Sousa & Hockings, 2015). Apart from bushmeat market studies in the capital (Sá et al., 2012; Minhós et al., 2013b), data north of the Corubal River, including Dulombi NP, only came from three surveys carried out more than twenty years ago (Limoges, 1989; Thibault, 1993; Gippoliti & Dell’Omo, 2003). In the Boké Region (Guinea) the most recent studies were carried out in the Sangarédi area (WCF, 2012; WCF, 2015a; WCF, 2015b). The only available data along the Guinea-Guinea-Bissau border came from a survey conducted in 1997 (Ham, 1998). Fifty percent (*N* = 37) of the studies from southern Senegal came from the long-term research site of Fongoli (McGrew, Pruetz & Fulton, 2005; Pruetz, 2006; Pruetz, 2007; Pruetz et al., 2015). The remaining studies mostly fell within the Niokolo-Koba NP (Galat et al., 2000; Galat, Galat-Luong & Nizinski, 2009), particularly Mt. Assirik (McGrew, Baldwin & Tutin, 1981; McGrew, Baldwin & Tutin, 1988; Baldwin, McGrew & Tutin, 1982; Harrison, 1983; Pruetz et al., 2012).

### Surveys in Dulombi National Park, Guinea-Bissau

During our surveys we detected seven primate species (Table 1). Campbell’s monkeys (*Cercopithecus campbelli*) were the most frequently encountered primate, ranging within riparian forest, open forest and woodland. We observed patas (*Erythrocebus patas*) and green monkeys (*Chlorocebus sabaeus*) using savannah grassland and woodland, as well as cashew (*Anacardium occidentale*) orchards. Red colobus and king colobus were mainly associated with riparian forests, whereas similarly to Campbell’s monkeys, chimpanzees occurred across riparian forests, open forests and savannah-woodland. Chimpanzees and red colobus were only recorded near Paiai Lumba. We heard one group of Guinea baboons (*Papio papio*) south of Paiai Lumba, and camera traps recorded one baboon group of c. 140 detected individuals in a riverine forest.

**Table 1:** Encounter rate of primate groups (sighted or heard) during recce walks and habitat type in which primates were observed in Dulombi National Park.

Species	IUCN Status	Dulombi (groups km^−1^)	Paiai (groups km^−1^)	Habitats
West African chimpanzee *Pan troglodytes verus*	CR	–	0.03	Riverine forest (obs, ct) Woodland (obs)
Temminck’s red colobus *Piliocolobus badius temminckii*	EN	–	0.07	Riverine forest (obs)
King colobus *Colobus polykomos*	VU	0.33	0.04	Agriculture-forest edge (obs) Riverine forest (obs, ct)
Guinea baboon *Papio papio*	NT	–	0.01	Riverine forest (obs, ct)
Green monkey *Chlorocebus sabaeus*	LC	0.23	0.10	Riverine forest (ct) Open forest (obs, ct) Woodland-grassland (obs) Agriculture (ct)
Campbell’s monkey *Cercopithecus campbelli*	LC	0.38	0.18	Riverine forest (obs, ct) Open forest (obs, ct)
Patas monkey *Erythrocebus patas*	LC	0.05	0.08	Woodland-grassland (obs) Agriculture (obs, ct)
Senegal galago *Galago senegalensis*	LC	NA	NA	Woodland (ct)

**Notes.**

Obs observed during recces ct detected by camera traps Survey effort 16 km of recces in Dulombi and 76 km in Paiai

Chimpanzee signs included 31 nest sites (*N* of nests = 66, average of 0.41 nest sites km^−1^ in Paiai), 18 clusters of feeding traces, dung remains and footprints all in the forest areas near Paiai. Nearly all chimpanzee nest sites were recorded within the riparian forest (30 of 31), with significantly more nests recorded on oil-palm trees than other tree species (47 of 66, *χ*^2^ = 124.9, *df* = 4, *p* < 0.001). Other tree species used to build nests were the *Parinari excelsa* (*N* = 10), *Dialium guineense* (*N* = 2), *Ficus ovata* (*N* = 2), *Detarium senegalense* (*N* = 1) and *Pterocarpus santalinoides* (*N* = 1). We also observed two chimpanzee nests built across two trees: one nest was built over an oil-palm and a *Ficus ovata* tree, and the second was built across two oil-palms.

### Primate occurrence across the region

Based on our literature search, chimpanzees were confirmed present in seven protected areas in Guinea-Bissau, one in Senegal and three in the Boké Region in Guinea (Table 2). Red colobus were observed in nine protected areas in Guinea-Bissau, one in Senegal and two in Guinea. King colobus were reported in seven protected areas in Guinea-Bissau and none in Boké Region (Guinea) nor Senegal.

**Table 2:** Most recent observations of threatened primates within protected areas in Guinea-Bissau, southern Senegal and Boké Region, Guinea, as reported in the literature. Sources: (1) Casanova & Sousa, 2007; (2) Limoges, 1989; (3) Carvalho, Marques & Vicente, 2013; (4) Chardonnet, 1983; (5) Hoogveld, 2013; (6) Kühl et al., 2016; (7) Oosterlynck & Wit, 2014; (8) Thibault, 1993; (9) Bessa, Sousa & Hockings, 2015; (10) Minhós et al., 2016; (11) Bailo, Alphonse & Gu, 2009; (12) Ham, 1998; (13) Galat, Galat-Luong & Nizinski, 2009; (14) Carter, 2000; (15) Sunderland-Groves et al., 2011; (16) Pruetz et al., 2012.

Region	Protected area	Chimpanzee	Temminck’s red colobus	King colobus	Source
Guinea-Bissau	Rio Grande de Buba	2005–2007	2005–2007	2005–2007	1
	Cufada NP	2011	1989	2005–2007	1,2,3
	Dulombi NP	2015 (1989)	2015 (1989)	2015 (1981)	This study (2,4)
	Tchétché corridor	1989	1989	–	2
	Boé NP	2014	2014	2012	5,6,7
	Cuntabane corridor	1993	1989	–	2,8
	Cantanhez NP	2013	2010	2010	9,10
	Salifo corridor	–	1989	1989	2
	Canquelifa Forest Reserve	–	1989	–	2
	Rio Cacheu Natural Park/ Pelundo Faunal Reserve	–	–	1989	2
Boké Region, Guinea	Badiar NP	–	2009	–	11
	N’Dama Classified Forest	1996–1997	Prior to 1997	–	12, 13
	Nialama Classified Forest	2008	–	-	14,15
	Tomine Koumba/Fello Digue Classified Forest	1996–1997	–	-	12
Southern Senegal	Niokolo-Koba NP	2012	2002	–	13,16

**Figure 4:**
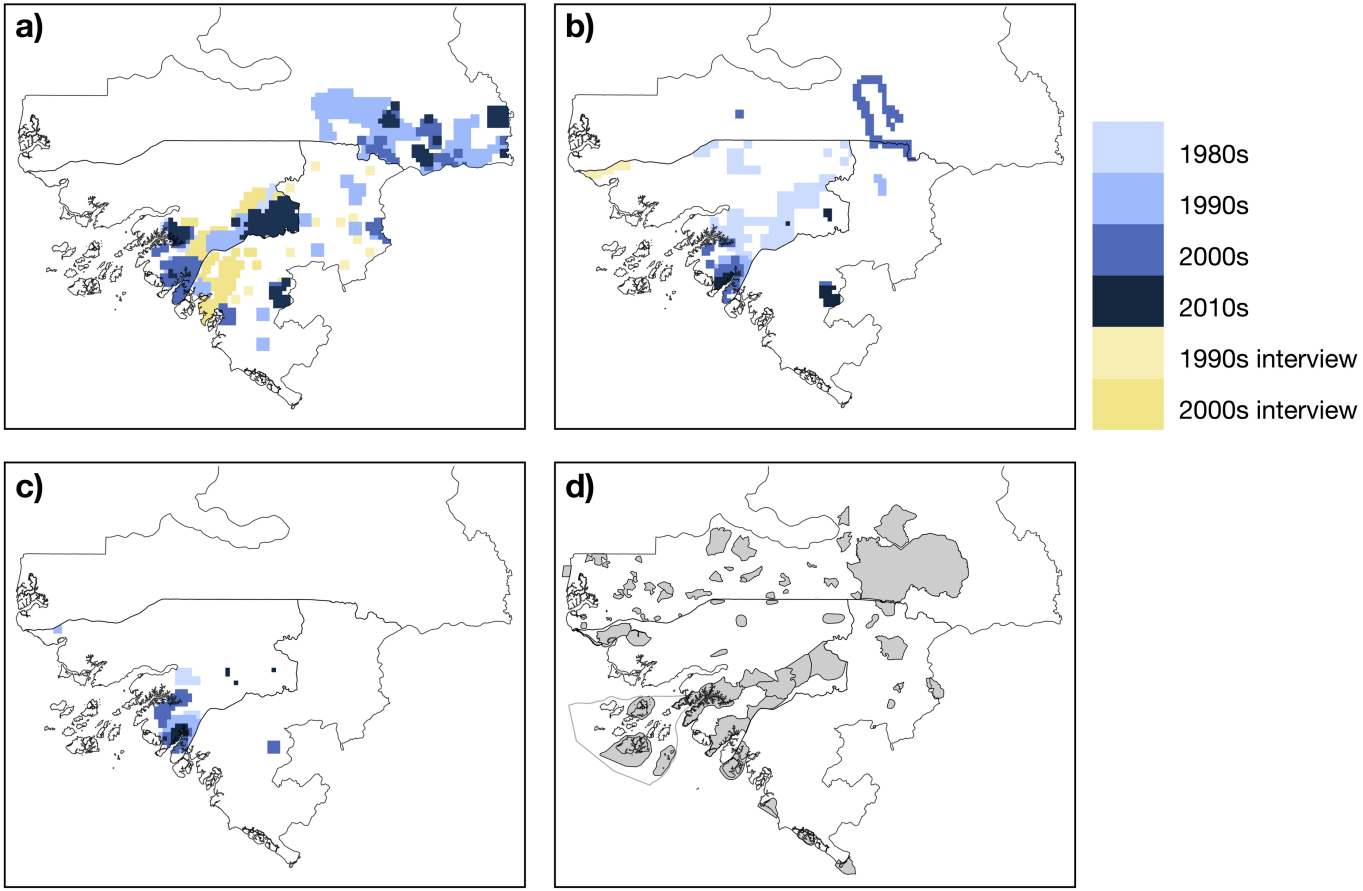
Mapped records of chimpanzee (A), red colobus (B) and king colobus (C) from studies conducted during the past thirty years, and map showing protected areas (D). Mapped records include data from published and unpublished material. Protected areas are taken from (UNEP-WCMC & IUCN, 2018).

In Senegal, chimpanzees were reported east of the Koulountou River, therefore in the southeast portion of the country including within, south and east of Niokolo-Koba NP (Fig. 4). In Guinea-Bissau chimpanzees were reported to occur across the majority of areas south of the Corubal River. North of the Corubal River they were confirmed present in Dulombi NP and were reported to occur between the Boé Sector and Gabú town (Limoges, 1989; Brugiere et al., 2009). In the Boké Region (Guinea), chimpanzees were recently surveyed in the Nialama Classified Forest (Carter, 2000; Sunderland-Groves et al., 2011) and Sangarédi (WCF, 2015a; WCF, 2015b; Kühl et al., 2016). Along the border with Guinea-Bissau, records of chimpanzees came from near Sansalé and Moyerai, i.e., close to the Cacine Basin and the Boé Sector in Guinea-Bissau, respectively (Ham, 1998).

In Senegal red colobus were recorded along the Koulountou and Gambia Rivers, and one group was observed within the town of Kolda (Galat, Galat-Luong & Nizinski, 2009). In Boké (Guinea), the species was only recorded in Badiar NP, along the Senegalese border, the N’Dama forest and Sangarédi (Bailo, Alphonse & Gu, 2009; Galat, Galat-Luong & Nizinski, 2009; WCF, 2015b). Red colobus were reported across southern Guinea-Bissau, with more recent studies coming from Cantanhez NP. Red colobus were also sighted in the northeastern parts of the country (Limoges, 1989). We found no study reporting the occurrence of king colobus in Senegal. In Guinea-Bissau, king colobus have been observed in the northwest part of the country close to the Senegalese border (Gippoliti & Dell’Omo, 2003). Most of the studies on king colobus were conducted in Cantanhez NP (Minhós et al., 2013a; Minhós et al., 2016). We found only one unpublished report noting the presence of this species in the Boé Sector in Guinea-Bissau (Hoogveld, 2013). Our study confirms the occurrence of king colobus in Dulombi NP. In Boké (Guinea), king colobus have been recorded in Sangarédi (Eriksson & Kpoghomou, 2006).

## Discussion

Our literature review highlights large geographical research gaps, particularly in southwestern Senegal (Casamance), northern Guinea-Bissau and the Boké Region (Guinea). A large proportion of information was retrieved from technical reports and conference abstracts. Had these documents not been made available online or published as supplementary journal issues, a large amount of data would have been missed, highlighting the importance of disseminating results to the wider scientific community. Although we might not have retrieved all unpublished research on primates in the region, reports on the presence of these threatened species are often outdated and spatially fragmented. There are surprisingly few data from Badiar NP, one of the only two NPs in Guinea, and despite the Boké Region (Guinea) being previously highlighted as a key biodiversity area (Brugiere & Kormos, 2009), primatological studies are sparse and out-of-date. In southwestern Senegal, there are 30 areas designated for protection from which no primatological data are available. We imagine that the reasons for these large geographical research gaps are not simply due to a lack of scientific interest. For example, the Casamance has seen over three decades of civil conflict, which ended with a ceasefire only relatively recently, in May 2014. Political instability, and logistical disadvantages such as the absence of a field station and/or considerable fieldwork costs, are likely to have dissuaded scientists from investing research efforts in certain areas. Similarly in Angola, it was only after the end of the civil conflict in 2002 that biologists began conducting research in the country (Ryan et al., 2004; Bersacola, Svensson & Bearder, 2015). When cost and time are the only constraints, even short and exploratory studies may provide biological information that can significantly contribute to a wider dataset. For example, brief surveys in Angola provided essential data on vocalisations which contributed to distinguishing and describing a new primate species (Svensson et al., 2017). Information from unsurveyed geographical areas in Guinea-Bissau and neighbouring countries could provide critical data for better understanding primate distributions and their conservation status, particularly for king colobus and red colobus. Indeed, through our preliminary surveys, we confirmed the presence of eight species of primates in Dulombi NP, including seven diurnal and one nocturnal species. Importantly, we confirmed the occurrence of the three most threatened primates in this region: the western chimpanzee, Temminck’s red colobus, and king colobus. Below we discuss our findings in the context of the available information about the three threatened primates’ geographical occurrence, and provide recommendation for planning future research and conservation strategies in Guinea-Bissau and neighbouring regions.

Similarly to other sites in Guinea-Bissau (e.g., Cantanhez, (Sousa et al., 2011), chimpanzees in Dulombi NP showed a preference for nesting on oil palms in riverine forests. In addition to nesting, oil palms are an important source of food for chimpanzees (Humle & Matsuzawa, 2004; Bessa, Sousa & Hockings, 2015; Bryson-Morrison, Matsuzawa & Humle, 2016). In Dulombi, oil palms are generally found in association with riverine and open forests. Future systematic surveys that account for variations in detection probability should be able to determine to what extent chimpanzees rely on riparian forests at this site. When planning future studies on chimpanzees, researchers should take into account complex issues in methods involving nest counting in areas where oil palm nests are predominant. As demonstrated by Sousa and colleagues (2011), the decay rate of oil palm nests is different from those built using other tree species, and varies depending on the type of nest (i.e., whether leaves are broken or folded by chimpanzees when making the nest). Due to logistical constraints, including extremely difficult access due to flooded roads, we were unable to visit other locations in Dulombi NP. Future studies in Dulombi NP should be carried out during the dry season and after the grassland is burned (Temudo, Figueira & Abrantes, 2015), therefore from February to May.

Geographical research gaps for chimpanzees in Guinea-Bissau are limited to areas north of the Corubal river in the Bafata region and around Canjadude in Boé. However, up-to-date surveys across the unprotected anthropogenic coastal forests in the Fulacunda, Empada and Catió peninsulas, as well as in the savannah-riparian forest mosaics of the Salifo, Tchétché and Cuntabane corridors will be useful to determine population connectivity and understand dynamics of chimpanzee coexistence with local people. In southeastern Senegal, the distribution of chimpanzees appears to be relatively homogeneous covering Niokolo-Koba NP and areas in the Kedougou region. Although historical records combined with recent studies suggest that chimpanzees are present throughout the Boké Region (Guinea), the occurrence of this species in Badiar NP remains unclear. In addition, up-to-date surveys should be carried out along the border with Guinea-Bissau, while in-depth biosocial studies in border areas could highlight cross-boundary interactions between people and chimpanzees.

The occurrence of red colobus in Dulombi NP suggests possible connectivity between the populations south of the Corubal River (which include Cufada, Cantanhez and Boé NP’s) and the presumed populations in the northern parts of the country. However, the current status of red colobus in north-western Guinea-Bissau remains uncertain, as the latest information was acquired nearly three decades ago (Limoges, 1989). The large geographical research gaps for red colobus in Guinea-Bissau include the entire coastal area northwest of the country (Kassolol, Pelundo Faunal Reserve, Cantchungu, Bissorã and Quinhàmel), and the central and northern areas (including Mansoa Forest Reserve and Dungal Forest Reserve near the border with Senegal, Supplemental Information 2). In Senegal, populations of red colobus were described as small and highly fragmented, including two isolated populations: one in the Saloum Delta NP (north of The Gambia, outside the geographical scope of this paper) and one in Niokolo-Koba NP (Galat, Galat-Luong & Nizinski, 2009). Information from interview-based surveys in Senegal carried out in 1998–1999 (Galat, Galat-Luong & Nizinski, 2009) suggests that red colobus may be absent from 13 areas designated for protection in southwestern Senegal. Up-to-date surveys across the Casamance and north-western Guinea-Bissau are crucially needed to establish the level of population fragmentation between the River Gambia and River Corubal, i.e., between the Gambian red colobus population and that in Dulombi NP. In Guinea, red colobus were surprisingly sighted in the Sangarédi area (WCF, 2015b). These observations extend the known eastern limits of the species’ geographical range (Galat-Luong et al., 2016) of at least 40 km. To date, it remains unclear whether red colobus are present in the fragmented coastal forests in southern Boké (Guinea) near the Bissau-Guinean border, and where the southeastern limit of the species’ range exactly is. In addition, up-to-date surveys in the tri-border area (including northeastern Guinea-Bissau, northwestern Guinea and southeastern Senegal) will be useful to determine the levels of (un)connectivity between the Niokolo-Koba and the Guinean/Bissau-Guinean populations.

King colobus in Dulombi NP was present in riverine forests and near agricultural fields. This species has mainly been described in forest habitats and some data suggest that they are negatively affected by forest fragmentation (Oates, Gippoliti & Groves, 2008; Minhós et al., 2016). The ability of king colobus to persist in the savannah-riverine forest anthropogenic mosaics in Dulombi indicates some level of ecological flexibility in this species. However, the rarity of this species’ occurrence in parts of its range (Limoges, 1989), its smaller average group sizes compared to sympatric primates (Davies & Oates, 1994) in addition to human activities, such as hunting and habitat modification, may make some populations under considerable pressure, which may not be necessarily evident at first sight. Like red colobus, in Cantanhez habitat fragmentation and hunting have caused a demographic bottleneck (Minhós et al., 2016). The fact that in the 1980s no king colobus were sighted in the north-western parts of Guinea-Bissau, i.e., the coastal areas including the Pelundo (Rio Cacheu Natural Park) and Mansoã zones (Limoges, 1989), brings to question whether there is still a possibility of connectivity between the southern populations and the small groups sighted in 1994 near São Domingos in the northwest region (Gippoliti & Dell’Omo, 2003). In southwestern Senegal the occurrence of this species in Basse-Casamance NP and at least six other forests designated for protection remains to be determined. Although they are expected to occur along the Guinean coast (Oates, Gippoliti & Groves, 2008), which forms part of the same eco-region as Cantanhez, Cufada and Cacine, no study to date has confirmed this. Therefore, priority future surveys should be carried out across the northernmost part of the king colobus range, particularly in remaining coastal forests south of the Casamance River, northwestern Guinea-Bissau and across the southern parts of Boké, Guinea (Supplemental Information 2).

**Table 3:** Details of next steps for research and conservation management of primates in Guinea-Bissau and neighbouring regions.

Step	Type	Aim	Where
Research	Biological surveys (including presence/absence from recce surveys covering a larger geographical scale, and/or survey techniques that take into account detection probabilities and give measures of abundance)	To assess population fragmentation levels, identify important populations, gather inferences of ecological flexibility, model distributions accurately at a fine scale, identify conservation threats	See Supplemental Information 2. Areas include along the Kogon river and Guinea-Bissau border in Guinea, Boké’s coastal areas, northern Guinea-Bissau and the Casamance in southwestern Senegal
	Interdisciplinary (biosocial)	To assess the effects of major threats and understand the underlying mechanisms	
	Social anthropological	To understand local people’s perceptions and behaviours towards conservation and wildlife	
	Social anthropological	To gather knowledge on the current environmental governance and land use management systems at the local and regional level, taking into account the sociocultural context, and find ways to increase local support for conservation and involve local people in land use management planning	
Planning and strategy	Revise, develop and implement land use spatial plans (e.g., protected landscape areas including zoning at the national, trans-national and local levels)	To reduce deforestation and improve conservation of important forest ecosystems and their corridors. To restore biological corridors allowing species movements	Across the region by the government and environmental agencies and at the local level with the involvement of local people
Strategy	Increase public environmental awareness on laws and regulations, management systems, and the importance of biodiversity	To promote environmentally friendly behaviour across the region and reduce illegal activities, particularly hunting and logging	Across the region, particularly in areas near the border
	Provide training and increase capacity for law enforcement	To reduce trans-boundary commercial illegal activities, particularly illegal bushmeat/pet trade and logging	At border control points, within protected areas

In light of the available data suggesting a likely fragmented population of red colobus across many parts of its range, and a possible fragmented population of king colobus in the northern regions we recommend that: (i) surveys are urgently carried out across the geographical areas indicated above (but see table in Supplemental Information 2 for details) to identify key populations and assess levels of population fragmentation, and (ii) together with chimpanzees, red colobus and king colobus should be considered among the top priority species when developing national and regional conservation policies and land use plans. Table 3 outlines the different stages to improve primate conservation management in Guinea-Bissau and neighbouring regions. Management strategies should take into account the anthropogenic nature of the landscape, which is typical across West Africa. For example, in Guinea-Bissau the majority of protected areas include high population density (e.g., over 100 villages are inside Cantanhez NP), as well as high biological diversity (IBAP, 2018). Therefore similarly to European models, many PA’s in Guinea-Bissau follow IUCN’s Category V (A Regalla, pers. comm., 2017), i.e., landscapes managed with the aim of maintaining or restoring sustainable interactions between people and nature (IUCN, 2016). Divided into a mosaic of management zones, e.g., protected forest, buffer, and human development zones, the protected landscape can play a crucial role in maintaining biodiversity, particularly when connectivity between the remaining forests is actively considered. Systematic monitoring in key ecological areas will be necessary for providing robust data on wildlife population trends, as well as indentifying changes in local conservation pressures/threats over time. Such monitoring programs will represent an essential tool to inform conservation policy from a biological perspective. In these agroforest landscapes, management objectives will need to focus on improving synergies between biodiversity conservation and agricultural development (Scherr & McNeely, 2008). The use of cross-disciplinary research approaches will be crucial for developing realistic, culturally and socially appropriate conservation strategies (Parathian et al., 2018). Finally, to ensure good environmental governance and maximise the public’s compliance with conservation policies (e.g., zoning, new hunting regulations), management strategies will require full local participation (Andrade & Rhodes, 2012), using a coordinated, multi-stakeholder approach including governmental agencies, local authorities, farmers and other local groups’ representatives (Scherr & McNeely, 2008). The development and evaluation of conservation actions and policies must be undertaken using rigorous frameworks, for example the Open Standards for the Practice of Conservation (CMP, 2018).

## Conclusion

Assessing population status and planning conservation action is difficult when baseline information on species occurrence is unavailable. In this study, we highlighted large knowledge gaps on the distribution of two colobines in Guinea-Bissau and neighbouring regions, and a lack of recent data on the occurrence of chimpanzees in western Guinea. The paucity of up-to-date information is particularly evident for red colobus, considering its relatively small geographical range. In regions where landscapes are largely human-dominated, forest environments and human-wildlife coexistence dynamics can change relatively quickly. Understanding the mechanisms of species persistence in these type of landscapes is therefore crucial to ensure that conservation management fits their needs. We intend for our study to be used as a framework by conservation researchers and practitioners when planning future primate research and conservation strategies in this region.

##  Supplemental Information

10.7717/peerj.4847/supp-1 Supplemental Information 1 Literature entries

10.7717/peerj.4847/supp-2 Supplemental Information 2 List of geographic locations including protected or unprotected areas identified as priority for future primate surveys. Numbers in brackets under the Areas column only refer to protected areas Data source: UNEP - WCMC Protected Planet (2014–2018) Available at: http//:www.protectedplanet.net. ^1^(Galat, Galat-Luong & Nizinski, 2009) conducted surveys in 1975–2002 across southern Senegal but do not specify if they surveyed the particular location reported in this table. We therefore report it here as presence ‘Unknown’.

10.7717/peerj.4847/supp-3 Supplemental Information 3 PRISMA checklist

10.7717/peerj.4847/supp-4 Supplemental Information 4 PRISMA flow diagram

## Additional Information and Declarations

### Field Study Permissions

The following information was supplied relating to field study approvals (i.e., approving body and any reference numbers): Fieldwork was approved by the Instituto da Biodiversidade e das Áreas Protegidas (IBAP), Guinea-Bissau.

### Data Availability

The following information was supplied regarding data availability: The literature search documents are provided in a Supplemental File.
