# Supplementary material for: Primate occurrence across a human-impacted landscape in Guinea-Bissau and neighbouring regions in West Africa: using a systematic literature review to highlight the next conservation steps
Source: PeerJ. 2018 May 23;6:e4847. doi: 10.7717/peerj.4847 (PMC5970555; doi:10.7717/peerj.4847)
Supplement: Supplemental Information 1 [file peerj-06-4847-s001.docx]

SI1. List of literature entries

**Guinea-Bissau**

1. Amador RC. 2014. Local perceptions and attitudes towards biodiversity in the Lagoas de Cufada Natural Park (LCNP), Guinea-Bissau. PhD Thesis. Lisbon, Portugal: Universidade de Lisboa.
2. Amador R., Casanova C., Lee PC. 2011. Ethnic speeches on non-human primate bushmeat at Lagoas de Cufada Natural Park (LCNP), Guinea-Bissau. In: *Folia Primatologica*. Karger, 359–359.
3. Amador R., Casanova C., Lee P. 2015. Ethnicity and perceptions of bushmeat hunting inside Lagoas de Cufada Natural Park (LCNP), Guinea-Bissau. *J Primatol* 3:1–8.
4. Barata A., Casanova C., Lee PC. 2011. Influence of human activity on chimpanzee distribution in the Cantanhez Woodland National Park, Guinea-Bissau. In: *Folia Primatologica*. Karger, 358–358.
5. Bessa J., Sousa C., Hockings KJ. 2015. Feeding ecology of chimpanzees (*Pan troglodytes verus*) inhabiting a forest-mangrove-savanna-agricultural matrix at Caiquene-Cadique, Cantanhez National Park, Guinea-Bissau. *American Journal of Primatology* 77:651–665. DOI: 10.1002/ajp.22388.
6. de Boer M. 2013. *Intensity of chimpanzee activity*. Bissau, Guinea-Bissau: Chimbo.
7. Bout N., Ghiurghi A. 2013. *Guide des mammiferes du Parc National de Cantanhez, Guinée-Bissau*. Acção para o Desenvolvimento, Guinea-Bissau and Associazione Interpreti Naturalistici ONLUS.
8. Brugiere D., Badjinca I., Silva C., Serra A. 2009. Distribution of chimpanzees and interactions with humans in Guinea-Bissau and western Guinea, West Africa. *Folia Primatologica* 80:353–358. DOI: 10.1159/000259335.
9. Cá A. 2008. Estudos sobre caça e mercado de primatas em Tombali, Sul da Guiné-Bissau. MSc Thesis. Belo Horizonte, Brazil: Universidade Federal de Minas Gerais.
10. Carminatti Wenceslau JF. 2014. *Bauxite mining and chimpanzees population distribution, a case study in the Boé sector, Guinea-Bissau*. Bissau, Guinea-Bissau: Chimbo.
11. Carvalho JI da S. 2014. Conservation status of the endangered chimpanzee (*Pan troglodytes verus*) in Lagoas de Cufada Natural Park (Republic of Guinea-Bissau). PhD Thesis. Lisbon, Portugal: Universidade de Lisboa.
12. Carvalho J., Casanova C., Vicente L. 2011. Estimating the density and abundance of *Pan troglodytes verus* by line transect sampling, in Lagoas de Cufada Natural Park, Republic of Guinea-Bissau. In: *Folia Primatologica*. Karger, 393–393.
13. Carvalho JS., Marques TA., Vicente L. 2013a. Nesting patterns of chimpanzees in relation to human disturbance and vegetation characteristics at Lagoas de Cufada Natural Park, Republic of Guinea-Bissau. In: *Folia Primatologica*. Karger, 257–258.
14. Carvalho JS., Marques TA., Vicente L. 2013b. Population status of *Pan troglodytes verus* in Lagoas de Cufada Natural Park, Guinea-Bissau. *PLOS ONE* 8:e71527. DOI: 10.1371/journal.pone.0071527.
15. Carvalho JS., Meyer CF., Vicente L., Marques TA. 2015. Where to nest? Ecological determinants of chimpanzee nest abundance and distribution at the habitat and tree species scale. *American journal of primatology* 77:186–199.
16. Carvalho JS., Vicente L., Marques TA. 2015. Chimpanzee (*Pan troglodytes verus*) diet composition and food availability in a human-modified landscape at Lagoas de Cufada Natural Park, Guinea-Bissau. *International Journal of Primatology* 36:802–822.
17. Casanova C., Sousa C. 2007. *National action plan for the conservation of the chimpanzee, red western colobus and black and white western colobus monkey populations in Guinea-Bissau*. Bissau, Guinea-Bissau: Instituto da Biodiversidade e Áreas Protegidas.
18. Casanova C., Sousa C., Costa S. 2014. Are animals and forests forever? perceptions of wildlife at Cantanhez Forest National Park, Guinea-Bissau Republic. *Memória-Special issue in anthropology and environment. Sociedade de Geografia de Lisboa, Lisboa*:40.
19. Chardonnet B. 1983. Gestion de la faune sauvage africaine: proposition d’un projet en Guinee-Bissau. Maisons-Alfort, France: École Nationale Vétérinaire d’Alfort.
20. Costa SG. 2010. Social perceptions of nonhumans in Tombali (Guinea-Bissau, West Africa): a contribution to chimpanzee (*Pan troglodytes verus*) conservation. United Kingdom: University of Stirling.
21. Costa S., Casanova CC., Sousa C., Lee PC. 2013. The good, the bad and the ugly: perceptions of wildlife in Tombali (Guinea-Bissau, West Africa). *Journal of Primatology* 2.
22. Ferreira da Silva MJ. 2012. Hunting pressure and the population genetic patterns and sex-mediated dispersal in the Guinea Baboon in Guinea-Bissau. Cardiff, United Kingdom: Cardiff University.
23. Ferreira da Silva MJ., Casanova C., Godinho R. 2013. On the western fringe of baboon distribution: mitochondrial D-loop diversity of Guinea baboons (*Papio papio* Desmarest, 1820) (Primates: Cercopithecidae) in coastal Guinea-Bissau, western Africa. *Journal of Threatened Taxa* 5:4441–4450. DOI: 10.11609/JoTT.o3216.4441-50.
24. Ferreira da Silva MJ., Godinho R., Casanova C., Minhós T., Sá R., Bruford MW. 2014. Assessing the impact of hunting pressure on population structure of Guinea baboons (*Papio papio*) in Guinea-Bissau. *Conservation genetics* 15:1339–1355.
25. Ferreira da Silva MJ., Sá R., Minhós T., Sousa F., Godinho R., Vicente L., Sousa C., Casanova C., Bruford MW. 2009. Evidence of non-human primate skin commerce for traditional practices in Guinea Bissau (West Africa). *Folia Primatologica* 80:394–425.
26. Gippoliti S., Dell’Omo G. 1995. Status and conservation of the chimpanzee Pan troglodytes verus in Guinea-Bissau. *African primates* 1:3–5.
27. Gippoliti S., Dell’Omo G. 1996. Primates of the Cantanhez forest and the Cacine basin, Guinea-Bissau. *Oryx* 30:74–80.
28. Gippoliti S., Dell’Omo G. 2003. Primates of Guinea-Bissau, West Africa: Distribution and conservation status. *Primate Conservation* 19:73–77.
29. Gippoliti S., Sousa C. 2004. The chimpanzee, *Pan troglodytes*, as an ‘umbrella’ species for conservation in Guinea-Bissau, West Africa: Opportunities and constraints. *Folia Primatologica* 75:385–414.
30. Hockings KJ., Sousa C. 2012. Differential utilization of cashew—a low-conflict crop—by sympatric humans and chimpanzees. *Oryx* 46:375–381. DOI: 10.1017/S003060531100130X.
31. Hockings KJ., Sousa C. 2013. Human-chimpanzee sympatry and interactions in Cantanhez National Park, Guinea-Bissau: Current research and future directions. *Primate Conservation* 26:57–65. DOI: 10.1896/052.026.0104.
32. Hoogveld J. 2013. *Using field data collected by local people to expand the knowledge of a large chimpanzee (Pan troglodytes verus) population in the Boé region of Guinea Bissau*. Bissau, Guinea-Bissau: Chimbo.
33. Kopp GH., Da Silva MF., Fischer J., Brito JC., Regnaut S., Roos C., Zinner D. 2014. The influence of social systems on patterns of mitochondrial DNA variation in baboons. *International journal of primatology* 35:210–225.
34. Kühl HS., Kalan AK., Arandjelovic M., Aubert F., D’Auvergne L., Goedmakers A., Jones S., Kehoe L., Regnaut S., Tickle A., Ton E., van Schijndel J., Abwe EE., Angedakin S., Agbor A., Ayimisin EA., Bailey E., Bessone M., Bonnet M., Brazolla G., Buh VE., Chancellor R., Cipoletta C., Cohen H., Corogenes K., Coupland C., Curran B., Deschner T., Dierks K., Dieguez P., Dilambaka E., Diotoh O., Dowd D., Dunn A., Eshuis H., Fernandez R., Ginath Y., Hart J., Hedwig D., Ter Heegde M., Hicks TC., Imong I., Jeffery KJ., Junker J., Kadam P., Kambi M., Kienast I., Kujirakwinja D., Langergraber K., Lapeyre V., Lapuente J., Lee K., Leinert V., Meier A., Maretti G., Marrocoli S., Mbi TJ., Mihindou V., Moebius Y., Morgan D., Morgan B., Mulindahabi F., Murai M., Niyigabae P., Normand E., Ntare N., Ormsby LJ., Piel A., Pruetz J., Rundus A., Sanz C., Sommer V., Stewart F., Tagg N., Vanleeuwe H., Vergnes V., Willie J., Wittig RM., Zuberbuehler K., Boesch C. 2016. Chimpanzee accumulative stone throwing. *Scientific Reports* 6:22219. DOI: 10.1038/srep22219.
35. van Laar JWT. 2010. *A Rapid rural appraisal of the bushmeat market in the Boé region, Guinea-Bissau: An investigation of the interaction between fauna (and flora) and people in the Boé region, Guinea-Bissau*. Bissau, Guinea-Bissau: Chimbo.
36. Limoges B. 1989. *Résultats de l’inventaire faunique au niveau national et propositions de modifications à la loi sur la chasse*. Bissau, Guinea-Bissau: Républica da Guiné-Bissau Ministério do Desenvolvimento Rural e da Agricultura.
37. Minhós T. 2012. Socio-genetics and population structure of two African colobus monkeys in Cantanhez National Park, Guinea-Bissau. United Kingdom: Cardiff University.
38. Minhós T., Chikhi L., Sousa C., Vicente LM., Ferreira da Silva M., Heller R., Casanova C., Bruford MW. 2016. Genetic consequences of human forest exploitation in two colobus monkeys in Guinea Bissau. *Biological Conservation* 194:194–208. DOI: 10.1016/j.biocon.2015.12.019.
39. Minhós T., Nixon E., Sousa C., Vicente LM., da Silva MF., Sá R., Bruford MW. 2013a. Genetic evidence for spatio-temporal changes in the dispersal patterns of two sympatric African colobine monkeys. *American Journal of Physical Anthropology* 150:464–474. DOI: 10.1002/ajpa.22223.
40. Minhós T., Wallace E., Ferreira da Silva MJ., Sá RM., Carmo M., Barata A., Bruford MW. 2013b. DNA identification of primate bushmeat from urban markets in Guinea-Bissau and its implications for conservation. *Biological Conservation* 167:43–49. DOI: 10.1016/j.biocon.2013.07.018.
41. Oosterlynck B., Wit P. 2014. *The impact of agriculture on the biodiversity in the Boé region (Guinea Bissau)*. Bissau, Guinea-Bissau: Chimbo.
42. Reiner F., Simões AP. 1999. *Mamíferos selvagens da Guiné-Bissau*. Projecto Delfim, Centro Portuguûes de Estudos dos Mamíferos Marinhos.
43. Robillard MJ. 1989. *Study of the use and perception of Guinea-Bissau fauna and natural environment .* Bissau, Guinea-Bissau: Ministere du Developpement Rural et Agriculture, Bissau (Guinea-Bissau). Direction Generale des Services des Forets et de la Chasse.
44. Rosa F., Crespo MV., Mendes LF. 2002. Contribution to the knowledge of the fauna of the Lagoas de Cufada Natural Park. Parasite diversity in Cercopithecus mona campbelli (Mammalia; Primates)[Guinea-Bissau]. *Serie de Zoologia*.
45. Sá RMM. 2013. Phylogeography, conservation genetics and parasitology of chimpanzees (Pan troglodytes versus) in Guinea-Bissau, West Africa. PhD Thesis. Portugal: Universidade NOVA de Lisboa.
46. Sá R., Ferreira da Silva M., Sousa FM., Minhós T. 2012. The trade and ethnobiological use of chimpanzee body parts in Guinea-Bissau. *Traffic Bulletin* 24.
47. Sá RM., Petrášová J., Pomajbíková K., Profousová I., Petrželková KJ., Sousa C., Cable J., Bruford MW., Modrỳ D. 2013. Gastrointestinal symbionts of chimpanzees in Cantanhez National Park, Guinea-Bissau with respect to habitat fragmentation. *American Journal of Primatology* 75:1032–1041.
48. Serra A., Silva C., Lopes E. 2007. *Étude de la faisabilité du projet “Developpément touristique de la Boé au profit de la conservation des Chimpanzés et des populations locales.”* Bissau, Guinea-Bissau.
49. Sousa FM. 2009. Densidade de *Pan troglodytes verus* e uso de recursos naturais pela população local, (Gadamael, República da Guiné-Bissau). MSc Thesis. Lisbon, Portugal: Universidade de Lisboa.
50. Sousa J., Barata AV., Sousa C., Casanova CCN., Vicente L. 2011. Chimpanzee oil-palm use in southern Cantanhez National Park, Guinea-Bissau. *American Journal of Primatology* 73:485–497. DOI: 10.1002/ajp.20926.
51. Sousa J., Casanova C., Barata AV., Sousa C. 2014a. The effect of canopy closure on chimpanzee nest abundance in Lagoas de Cufada National Park, Guinea-Bissau. *Primates* 55:283–292.
52. Sousa C., Frazão-Moreira A. 2010. Etnoprimatologia ao serviço da conservação na Guiné-Bissau: o chimpanzé como exemplo. *Etnoecologia em Perspectiva: natureza, cultura e conservação*.
53. Sousa J., Vicente L., Gippoliti S., Casanova C., Sousa C. 2014b. Local knowledge and perceptions of chimpanzees in Cantanhez National Park, Guinea-Bissau. *American Journal of Primatology* 76:122–134.
54. Thibault M. 1993. *Parc National de Dulombi: Bilan des inventaires de mammifères de 1990 à 1993 et potentiel d’exploitation.* Bafatá, Guinea-Bissau: CECI.
55. Thomas N., Sa RM., Ferreira da Silva M., Minhos T., Bruford MW. 2011. Mitochondrial DNA variation of the lesser spot-nosed guenon (*Cercopithecus petaurista*) from Canhabaque Island, Bijagos Archipelago, West Africa. In: *Folia Primatologica*. Karger, 396–397.
56. Torres J., Brito JC., Vasconcelos MJ., Catarino L., Gonçalves J., Honrado J. 2010. Ensemble models of habitat suitability relate chimpanzee (*Pan troglodytes*) conservation to forest and landscape dynamics in Western Africa. *Biological Conservation* 143:416–425. DOI: 10.1016/j.biocon.2009.11.007.

**Southern Senegal**

1. Anderson JR., McGrew WC. 1984. Guinea baboons (*Papio papio*) at a sleeping site. *American Journal of Primatology* 6:1–14. DOI: 10.1002/ajp.1350060102.
2. Baldwin PJ., McGrew WC., Tutin CEG. 1982. Wide-ranging chimpanzees at Mt. Assirik, Senegal. *International Journal of Primatology* 3:367–385. DOI: 10.1007/BF02693739.
3. Baldwin PJ., Pí JS., McGrew WC., Tutin CEG. 1981. Comparisons of nests made by different populations of chimpanzees (*Pan troglodytes*). *Primates* 22:474–486. DOI: 10.1007/BF02381239.
4. Bermejo M., Illera G., Sabater-Pí J. 1989. New observations on the tool-behavior of chimpanzees from Mt. Assirik (Senegal, West Africa). *Primates* 30:65–73. DOI: 10.1007/BF02381211.
5. Bertolani P., Pruetz JD. 2011. Seed reingestion in savannah chimpanzees (*Pan troglodytes verus*) at Fongoli, Senegal. *International Journal of Primatology* 32:1123. DOI: 10.1007/s10764-011-9528-5.
6. Bogart SL., Pruetz JD. 2008. Ecological context of savanna chimpanzee (*Pan troglodytes verus)* termite fishing at Fongoli, Senegal. *American Journal of Primatology* 70:605–612. DOI: 10.1002/ajp.20530.
7. Bogart SL., Pruetz JD. 2009. Savanna chimpanzee (*Pan troglodytes verus*) feeding ecology at Fongoli, Senegal. In: *American Journal of Physical Anthropology*. Wiley, 95–95.
8. Bogart SL., Pruetz JD. 2011. Insectivory of savanna chimpanzees (*Pan troglodytes verus*) at Fongoli, Senegal. *American Journal of Physical Anthropology* 145:11–20. DOI: 10.1002/ajpa.21452.
9. Bogart SL., Pruetz JD., McGrew WC. 2005. Termite de jour: termite “fishing” by West African chimpanzees (*Pan troglodytes verus*) at Fongoli, Senegal. In: *American Journal of Physical Anthropology*. Wiley, 75–75.
10. Bogart S l., Pruetz J d., Ormiston L k., Russell J l., Meguerditchian A., Hopkins W d. 2012. Termite fishing laterality in the Fongoli savanna chimpanzees (*Pan troglodytes verus*): Further evidence of a left hand preference. *American Journal of Physical Anthropology* 149:591–598. DOI: 10.1002/ajpa.22175.
11. Boyer KM. 2011. Chimpanzee conservation in light of impending iron ore mining project in SE Senegal. PhD Thesis. United States: Iowa State University.
12. Boyer KM., Pruetz JD. 2011. The effects of human disturbance on chimpanzee (*Pan troglodytes verus)* nesting in the mining zone of Senegal prior to iron mine construction. In: *American Journal of Primatology*. Wiley, 55–55.
13. Boyer-Ontl KM., Pruetz JD. 2014. Giving the forest eyes: The benefits of using camera traps to study unhabituated chimpanzees (*Pan troglodytes verus*) in Southeastern Senegal. *International Journal of Primatology* 35:881–894. DOI: 10.1007/s10764-014-9783-3.
14. Byrne RW. 1981. Distance vocalisations of Guinea baboons (*Papio papio*) in Senegal: an analysis of function. *Behaviour* 78:283–312. DOI: 10.1163/156853981X00365.
15. Cisse MG., Pruetz JD. 2009. Behavioural features of baobab smashing by savannah chimpanzees (*Pan troglodytes verus*) at Fongoli, Senegal: A referential model for the origin of human material culture. In: *Folia Primatologica*. Karger, 113–113.
16. Di Silvestre I., Novelli O., Bogliani G. 2000. Feeding habits of the spotted hyaena in the Niokolo Koba National Park, Senegal. *African Journal of Ecology* 38:102–107. DOI: 10.1046/j.1365-2028.2000.00220.x.
17. Ebbert MA., McGrew WC., Marchant LF. 2013. Community composition, correlations among taxa, prevalence, and richness in gastrointestinal parasites of baboons in Senegal, West Africa. *Primates* 54:183–189. DOI: 10.1007/s10329-012-0339-x.
18. Ebbert MA., McGREW WC., Marchant LF. 2015. Differences between chimpanzee and baboon gastrointestinal parasite communities. *Parasitology* 142:958–967. DOI: 10.1017/S0031182015000104.
19. Galat G., Galat Luong A., Ndiaye L., Keita Y. 2000. Geographical distribution of chimpanzees and baboons in Senegal. *Folia Primatologica* 71.
20. Galat G., Galat-Luong A., Nizinski G. 2009. Increasing dryness and regression of the geographical range of Temminck’s red colobus *Procolobus badius temminckii*: implications for its conservation. *Mammalia* 73:365–368. DOI: 10.1515/MAMM.2009.051.
21. Galat Luong A., Galat G. 2000. Chimpanzees and baboons drink filtrated water. In: *Folia Primatologica*. Karger, 71.
22. Galat-Luong A., Galat G., Hagell S. 2006. The social and ecological flexibility of Guinea baboons: Implications for Guinea baboon social organization and male strategies. In: *Reproduction and Fitness in Baboons: Behavioral, Ecological, and Life History Perspectives*. Developments in Primatology: Progress and Prospects. Springer, Boston, MA, 105–121. DOI: 10.1007/978-0-387-33674-9_5.
23. Galat-Luong A., Galat G., Ndiaye I., Keita Y. 1999. Fragmentation de la distribution et statut actuel du chimpanzé *Pan troglodytes verus* en limite d’aire de répartition au Sénégal. *African Primates* 4:71–72.
24. Gandini G., Baldwin PJ. 1978. Encounter between chimpanzees and a leopard in Senegal. *Carnivore* 1:107–109.
25. Gaspersic M. 2011. The prospects for future co-existence of chimpanzees and humans in southeastern Senegal: A pilot study at Bandafassi. In: *Folia Primatologica*. Karger, 361–361.
26. Gaspersic M., Pruetz JD. 2008. Savanna chimpanzees (*Pan troglodytes verus*) and baobab fruits (Adansonia digitata): Investigation of percussive technology among three chimpanzee communities in southeastern Senegal. In: *Folia Primatologica*. Karger, 332–333.
27. Gaspersic M., Pruetz J. 2013. Savanna chimpanzees in Bandafassi Arrondissement, Senegal: The past and the future of sympatry with humans. In: *Folia Primatologica*. Karger, 275–275.
28. Harrison MJS. 1983a. Patterns of range use by the green monkey, *Cercopithecus sabaeus*, at Mt. Assirik, Senegal. *Folia Primatologica* 41:157–179. DOI: 10.1159/000156129.
29. Harrison MJS. 1983b. Age and sex differences in the diet and feeding strategies of the green monkey, *Cercopithecus sabaeus*. *Animal Behaviour* 31:969–977. DOI: 10.1016/S0003-3472(83)80001-3.
30. Harrison MJS. 1984. Optimal foraging strategies in the diet of the green monkey, *Cercopithecus sabaeus*, at Mt. Assirik, Senegal. *International Journal of Primatology* 5:435. DOI: 10.1007/BF02692269.
31. Harrison MJS. 1985. Time budget of the green monkey, *Cercopithecus sabaeus*: Some optimal strategies. *International Journal of Primatology* 6:351–376. DOI: 10.1007/BF02736383.
32. Henty CJ., McGrew WC. 2014. Ethology and ecology of the patas monkey (*Erythrocebus patas*) at Mt. Assirik, Senegal. *African Primates* 9:35–44.
33. Howells ME. 2007. Common origins: Commensalisms between humans and chimpanzees in southeastern Senegal. In: *American Journal of Physical Anthropology*. Wiley, 131–132.
34. Howells ME., Pruetz JD., Gillespie TR. 2006. Increased human-chimpanzee sympatry in southeastern Senegal: Implications for pathogen transmission and health. In: *American Journal of Physical Anthropology*. Wiley, 105–106.
35. Howells ME., Pruetz J., Gillespie TR. 2011. Patterns of gastro-intestinal parasites and commensals as an index of population and ecosystem health: The case of sympatric western chimpanzees (*Pan troglodytes verus*) and Guinea baboons (*Papio hamadryas papio*) at Fongoli, Senegal. *American Journal of Primatology* 73:173–179. DOI: 10.1002/ajp.20884.
36. Hunt KD., McGrew WC. 2002. Chimpanzees in the dry habitats of Assirik, Senegal and Semliki wildlife reserve, Uganda. In: *Behavioural diversity in chimpanzees and bonobos*. Cambridge, United Kingdom: Cambridge University Press, 35–51.
37. Kopp GH., Fischer J., Patzelt A., Roos C., Zinner D. 2015. Population genetic insights into the social organization of Guinea baboons (*Papio papio*): Evidence for female-biased dispersal. *American Journal of Primatology* 77:878–889. DOI: 10.1002/ajp.22415.
38. Lehmann J., Korstjens AH., Dunbar RIM. 2007. Fission–fusion social systems as a strategy for coping with ecological constraints: a primate case. *Evolutionary Ecology* 21:613–634. DOI: 10.1007/s10682-006-9141-9.
39. Lindshield SM., Danielson BJ., Pruetz JD. 2015. The ecology of fear and savanna resource limitation in western chimpanzees (*Pan troglodytes verus*) at Fongoli, Senegal. In: *American Journal of Physical Anthropology*. WILEY-BLACKWELL 111 RIVER ST, HOBOKEN 07030-5774, NJ USA, 205–205.
40. Lindshield S., Pruetz JD. 2013. Meat transfer among savanna chimpanzees at Fongoli, Senegal: The female perspective. In: *American Journal of Physical Anthropology*. WILEY-BLACKWELL 111 RIVER ST, HOBOKEN 07030-5774, NJ USA, 181–181.
41. McBeath NM., McGrew WC. 1982. Tools used by wild chimpanzees to obtain termites at Mt Assirik, Senegal: The influence of habitat. *Journal of Human Evolution* 11:65–72. DOI: 10.1016/S0047-2484(82)80032-8.
42. McGrew WC. 1983. Animal foods in the diets of wild chimpanzees (*Pan troglodytes*): Why cross-cultural variation? *Journal of Ethology* 1:46–61. DOI: 10.1007/BF02347830.
43. McGrew W., Baldwin P., Marchant L., Pruetz J., Scott S., Tutin C. 2002. Ethno-archaeology of unhabituated chimpanzees at Mont Assirik, Senegal, West Africa. In: *American Journal of Physical Anthropology*. WILEY-BLACKWELL 111 RIVER ST, HOBOKEN 07030-5774, NJ USA, 110–110.
44. McGrew WC., Baldwin PJ., Marchant LF., Pruetz JD., Tutin CEG. 2014. Chimpanzees (*Pan troglodytes verus*) and their mammalian sympatriates: Mt. Assirik, Niokolo-Koba National Park, Senegal. *Primates* 55:525–532. DOI: 10.1007/s10329-014-0434-2.
45. McGrew WC., Baldwin PJ., Tutin CEG. 1981. Chimpanzees in a hot, dry and open habitat: Mt. Assirik, Senegal, West Africa. *Journal of Human Evolution* 10:227–244. DOI: 10.1016/S0047-2484(81)80061-9.
46. McGrew WC., Baldwin PJ., Tutin CEG. 1988. Diet of wild chimpanzees (*Pan troglodytes verus)* at Mt. Assirik, Senegal: I. Composition. *American Journal of Primatology* 16:213–226. DOI: 10.1002/ajp.1350160304.
47. McGrew W c., Ensminger A l., Marchant L f., Pruetz J d., Vigilant L. 2004. Genotyping aids field study of unhabituated wild chimpanzees. *American Journal of Primatology* 63:87–93. DOI: 10.1002/ajp.20041.
48. McGrew WC., Johnson-Fulton S., Pruete JD. 2004. Elementary technology of the wild chimpanzees of Fongoli, Senegal. In: *American Journal of Physical Anthropology*. Wiley, 146–146.
49. McGrew WC., Pruetz JD., Fulton SJ. 2005. Chimpanzees use tools to harvest social insects at Fongoli, Senegal. *Folia Primatologica* 76:222–226. DOI: 10.1159/000086023.
50. McGrew WC., Tutin CEG., Baldwin PJ. 1979. Chimpanzees, tools, and termites: Cross-cultural comparisons of Senegal, Tanzania, and Rio Muni. *Man* 14:185–214. DOI: 10.2307/2801563.
51. Ndiaye PI., Galat G., Galat-Luong A., Nizinski G. 2013. Note on the seasonal use of lowland and highland habitats by the West African Chimpanzee *Pan troglodytes verus* (Schwarz, 1934) (Primates: Hominidae): Implications for its conservation. *Journal of Threatened Taxa* 5:3697–3700. DOI: 10.11609/JoTT.o3229.3697-700.
52. Ontl KB., Pruetz JD. 2016. Dynamics of human-chimpanzee encounters at Fongoli, Senegal, 2006-2014. In: *American Journal of Physical Anthropology*. Wiley, 100–101.
53. Piel A., Pruetz J. 2004. Scarce resources and party size: How chimpanzees adapt to a severe habitat in SE Senegal. In: *Folia Primatologica*. Karger, 317–317.
54. Pruetz J. 2002. Competition between savanna chimpanzees and humans in southeastern Senegal. In: *American Journal of Physical Anthropology*. Wiley, 128–128.
55. Pruetz JD. 2005. Cave use by wild savanna chimpanzees (*Pan troglodytes verus*) in Senegal: behavioral adaptation to heat stress? In: *American Journal of Physical Anthropology*. Wiley, 168–168.
56. Pruetz JD. 2006. Feeding ecology of savanna chimpanzees (*Pan troglodytes verus*) at Fongoli, Senegal. In: *Hohmann, G., Robbins, M.M., Boesch, C. (eds.) Feeding ecology in apes and other primates*. United Kingdom: Cambridge University Press, 326–364.
57. Pruetz JD. 2011. Targeted helping by a wild adolescent male chimpanzee (*Pan troglodytes verus*): Evidence for empathy? *Journal of Ethology* 29:365–368. DOI: 10.1007/s10164-010-0244-y.
58. Pruetz JD., Ballahira R., Camara W., Lindshield S., Marshack JL., Olson A., Sahdiako M., Villalobos-Flores U. 2012. Update on the Assirik chimpanzee (*Pan troglodytes verus*) population in Niokolo Koba National Park, Senegal. *Pan Africa News*.
59. Pruetz JD., Bertolani P. 2007. Savanna chimpanzees, *Pan troglodytes verus*, hunt with tools. *Current Biology* 17:412–417. DOI: 10.1016/j.cub.2006.12.042.
60. Pruetz JD., Bertolani P., Ontl KB., Lindshield S., Shelley M., Wessling EG. 2015. New evidence on the tool-assisted hunting exhibited by chimpanzees (*Pan troglodytes verus*) in a savannah habitat at Fongoli, Sénégal. *Open Science* 2:140507. DOI: 10.1098/rsos.140507.
61. Pruetz JD., Knutsen P. 2003. Scrambling for a common resource: Chimpanzees, humans, and *Saba senegalensis*, in southeastern Senegal. In: *American Journal of Physical Anthropology*. Wiley, 172–172.
62. Pruetz JD., LaDuke TC. 2010. Brief communication: Reaction to fire by savanna chimpanzees (*Pan troglodytes verus*) at Fongoli, Senegal: Conceptualization of “fire behavior” and the case for a chimpanzee model. *American Journal of Physical Anthropology* 141:646–650. DOI: 10.1002/ajpa.21245.
63. Pruetz JD., Lindshield S. 2012. Plant-food and tool transfer among savanna chimpanzees at Fongoli, Senegal. *Primates* 53:133–145. DOI: 10.1007/s10329-011-0287-x.
64. Pruetz J d., Marchant L f., Arno J., McGrew W c. 2002. Survey of savanna chimpanzees (*Pan troglodytes verus*) in southeastern Senegal. *American Journal of Primatology* 58:35–43. DOI: 10.1002/ajp.10035.
65. Pruetz JD., Marshack JL. 2009. Savanna Chimpanzees (*Pan troglodytes verus*) prey on patas monkeys (*Erythrocebus patas*) at Fongoli, Senegal. *Pan Africa News* 16:15–17.
66. Pruetz JD., McGrew WC., Marchant LF., Arno J. 2001. Status of the savanna chimpanzees (*Pan troglodytes verus*) at Mont Assirik in Parc National du Niokolo Koba and in adjacent areas in southeastern Senegal. In: *American Journal of Physical Anthropology*. Wiley, 121–121.
67. Pruetz J., Socha A., Kante D. 2010. New range record for the lesser spot-nosed guenon (*Cercopithecus petaurista*) in southeastern Senegal. *African Primates*:64–66.
68. Pruetz JD., Tourkakis CA., Lindshield S. 2009. Locomotion, posture and substrate use by West African chimpanzees (*Pan troglodytes verus*) in the savanna environment of Fongoli, Senegal. In: *American Journal of Primatology*. Wiley, 90–90.
69. Russak SM., McGrew W c. 2008. Chimpanzees as fauna: comparisons of sympatric large mammals across long-term study sites. *American Journal of Primatology* 70:402–409. DOI: 10.1002/ajp.20506.
70. Sharman MJ. 1982. Feeding, ranging and social organisation of the Guinea baboon. PhD Thesis. United Kingdom: University of St Andrews.
71. Skinner MF., Pruetz JD. 2012. Reconstruction of periodicity of repetitive linear enamel hypoplasia from perikymata counts on imbricational enamel among dry-adapted chimpanzees (*Pan troglodytes verus*) from Fongoli, Senegal. *American Journal of Physical Anthropology* 149:468–482. DOI: 10.1002/ajpa.22145.
72. Stewart F a. 2011. Brief communication: Why sleep in a nest? empirical testing of the function of simple shelters made by wild chimpanzees. *American Journal of Physical Anthropology* 146:313–318. DOI: 10.1002/ajpa.21580.
73. Stewart FA., Pruetz JD. 2013. Do Chimpanzee nests serve an anti-predatory function? *American Journal of Primatology* 75:593–604. DOI: 10.1002/ajp.22138.
74. Stewart FA., Pruetz JD., Hansell MH. 2007. Do chimpanzees build comfortable nests? *American Journal of Primatology* 69:930–939. DOI: 10.1002/ajp.20432.
75. Tutin CEG., McGrew WC., Baldwin PJ. 1981. Responses of wild chimpanzees to potential predators. In: *Primate Behavior and Sociobiology*. Proceedings in Life Sciences. Springer, Berlin, Heidelberg, 136–141. DOI: 10.1007/978-3-642-68254-4_19.
76. Tutin CEG., McGrew WC., Baldwin PJ. 1983. Social organization of savanna-dwelling chimpanzees, *Pan troglodytes verus*, at Mt. Assirik, Senegal. *Primates* 24:154–173. DOI: 10.1007/BF02381079.
77. Verschuren JC. 1982. Notes de bio-ecologie des grands mammiferes du Park National du Niokolo-Koba. Examen compare avec le Zaire et l’Afrique de l’Est. *Memoires de l’Institut Fondamental d’Afrique Noire* 92:233–278.
78. Wessling EG., Kühl HS., Deschner T., Pruetz JD. 2015. Constraints associated with living in a savannah-woodland environment: Seasonal stress patterns in chimpanzees (Pan troglodytes verus) at Fongoli, Senegal. In: *Folia Primatologica*. Karger, 380.

**Boké, Guinea**

1. Bailo DS., Alphonse N., Gu Y. 2009. An inventory of biodiversity in the Badiar National Park, Guinea Conakry: Implication for conservation. *Research Journal of Biological Sciences* 4:948–951.
2. Carter J. 2000. *Les chimpanzés de Guinée. Une etude pour une survie: Nyalama et Pita*. Conackry, Republic of Guinea: US Agency for International Development, the Direction Nationale des Eaux et Forets.
3. Eriksson J., Kpoghomou E. 2006. A Rapid Survey of the Primates of Boké Préfecture, Northwestern Guinea. In: *A Rapid Biological Assessment of Boké Préfecture, Northwestern Guinea*. Washington, USA: Conservation International, 146–151.
4. Ham R. 1998. *Nationwide chimpanzee survey and large mammal survey, Republic of Guinea*. Conackry, Republic of Guinea: European Communion.
5. Jankowski F., Joulian F. 2007. Interpreting baboon reactions during ‘habituation’: observer-primate relationship in the field. In: *Folia Primatologica*. Karger, 200–201.
6. Kormos R., Boesch C., Bakarr MI., Butynski TM. 2003. *West African chimpanzees: status survey and conservation action plan*. International Union for Conservation of Nature and Natural Resources.
7. Leblan V. 2007. Chimpanzees, *Pan troglodytes verus*, in anthropic environments: Ecology and ethno-history of their interactions with Fula and Landuma human communities of the Kakandé Region (Guinea). In: *Folia Primatologica*. Karger, 201.
8. Leblan V. 2008. Anthropological dimensions of spatial interactions between chimpanzees and humans: A case study from the Boke region, northwestern Guinea. In: *Folia Primatologica*. Karger, 349–349.
9. Leblan V. 2014. The impact of West African trade on the distribution of chimpanzee and elephant populations (Guinea, Guinea-Bissau, Senegal, 19th–20th century). *Human Ecology* 42:455–465. DOI: 10.1007/s10745-014-9654-8.
10. Leblan V. 2016. Territorial and land-use rights perspectives on human-chimpanzee-elephant coexistence in West Africa (Guinea, Guinea-Bissau, Senegal, nineteenth to twenty-first centuries). *Primates* 57:359–366. DOI: 10.1007/s10329-016-0532-4.
11. Leblan V., Bricka B. 2013. Genies or the opacity of human-animal relationships in Kakande, Guinea. *African Study Monographs* 34:85–108. DOI: 10.14989/179135.
12. Leciak E., Hladik A., HLADIK C-M. 2005. Le palmier à huile (*Elaeis guineensis*) et les noyaux de biodiversité des forêts-galeries de Guinée maritime: à propos du commensalisme de l’homme et du chimpanzé. *Revue d’Écologie* 2:179–184.
13. Sugiyama Y., Soumah AG. 1988. Preliminary survey of the distribution and population of chimpanzees in the Republic of Guinea. *Primates* 29:569–574. DOI: 10.1007/BF02381144.
14. Sunderland-Groves JL., Slayback DA., Balinga MPB., Sunderland TCH. 2011. Impacts of co-management on western chimpanzee (*Pan troglodytes verus*) habitat and conservation in Nialama Classified Forest, Republic of Guinea: A satellite perspective. *Biodiversity and Conservation* 20:2745. DOI: 10.1007/s10531-011-0102-4.
15. WCF. 2012. *Final annual report*. Wild Chimpanzee Foundation.
16. WCF. 2015a. *Final annual report*. Wild Chimpanzee Foundation.
17. WCF. 2015b. *Complementary primates study CBG expansion project - rapid assessment*. Wild Chimpanzee Foundation.
