# Supplementary material for: Primate occurrence across a human-impacted landscape in Guinea-Bissau and neighbouring regions in West Africa: using a systematic literature review to highlight the next conservation steps
Source: PeerJ. 2018 May 23;6:e4847. doi: 10.7717/peerj.4847 (PMC5970555; doi:10.7717/peerj.4847)
Supplement: Supplemental Information 2 — Data source: UNEP - WCMC Protected Planet (2014–2018) Available at: http//:www.protectedplanet.net. 1(Galat, Galat-Luong & Nizinski, 2009) conducted surveys in 1975–2002 across southern Senegal but do not specify if they surveyed the particular location reported in this table. We therefore report it here as presence ‘Unknown’. [file peerj-06-4847-s002.docx]

SI.2 List of geographic locations including protected or unprotected areas identified as priority for future primate surveys. Numbers in brackets under the Areas column only refer to protected areas. Data source: UNEP - WCMC Protected Planet (2014-2018) Available at: www.protectedplanet.net

| # | Areas | Size (km^2^) | IUCN Category | Habitats included in landscape | Surveys needed for | Present? |
| --- | --- | --- | --- | --- | --- | --- |
|  | Senegal, southwest |  |  |  |  |  |
| 1 | (1) Narangs and  (2) Essom Forest Reserves | 244 | NR | Open forest, woodland, grassland, villages, agriculture, roads | Temminck’s red colobus | Unknown |
| 2 | Tendouck, southwest of Diégoun | NA | NA | Forest, open forest, agriculture | Temminck’s red colobus | Unknown |
| 3 | (3) Kandiadiou,  (4) Koulayae,  (5) Djipakoum,  (6) Kalounayes,  (7) Bignona and  (8) Tobor Forest Reserves | 340 | NA | Open forest, woodland, villages, agriculture, roads | Temminck’s red colobus | Unknown |
| 4 | (9) Yassine,  (10) Bari,  (11) Boundie,  (12) Balmadou and  (13) Mangaroungou Forest Reserves | 588 | NR | Woodland, grassland, roads | Temminck’s red colobus | Unknown |
| 5 | (14) Pata Forest Reserve | 641 | NR | Open forest/woodland, roads, villages, dominated by agriculture | Temminck’s red colobus | Unknown^1^ |
| 6 | (15) Guimara Forest Reserve | 498 | NR | Woodland, agriculture, villages, roads | Temminck’s red colobus | Unknown^1^ |
| 7 | (16) Sadiata and  (17) Diatouma Forest Reserves | 143 | NR | Woodland, roads | Temminck’s red colobus | Unknown^1^ |
| 8 | (18) Mahon and  (19) Bakor Forest Reserves | 195 | NR | Woodland, riverine forest | Temminck’s red colobus | Unknown, but reported in adjacent Kolda town by Galat et al (2009) |
| 9 | (20) Dabo and  (21) Toutoune Forest Reserves | 153 | NR | Woodland, roads, agriculture | Temminck’s red colobus | Unknown^1^ |
| 10 | (22) Koudora Forest Reserve | 61 | NR | Open forest, woodland | Temminck’s red colobus | Unknown^1^ |
| 11 | (23) Diantene,  (24) Oukout and  (25) Boukitingo Forest Reserves | 24 | NR | Forest, woodland, logged forest, roads | King colobus  Temminck’s red colobus | Unknown  Unknown |
| 12 | (26) Basse-Casamance National Park | 50 | II | Forest, open forest, mangroves, roads | King colobus  Temminck’s red colobus | Unknown  Unknown |
| 13 | (27) Djibelor Forest Reserve | 2 | NR | Forest, mangroves, roads | King colobus  Temminck’s red colobus | Unknown  Unknown |
| 14 | (28) Bayot Forest Reserve | 13 | NR | Forest, woodland, logged forest, roads | King colobus  Temminck’s red colobus | Unknown  Unknown |
| 15 | (29) Bissine and Biaz Forest Reserve | 87 | NR | Open forest, woodland, riverine forest, logged forest, roads, villages, agriculture | King colobus  Temminck’s red colobus | Unknown  Unknown |
| 16 | (30) Bafata Forest Reserve | 32 | NR | Woodland, open forest, roads, agriculture | King colobus  Temminck’s red colobus | Unknown  Unknown |
|  |  |  |  |  |  |  |
|  | Guinea-Bissau |  |  |  |  |  |
| 17 | Kassolol, east of Varela | NA | NA | Forest, agriculture, roads, villages | King colobus  Temminck’s red colobus | Both species unsighted by Limoges (1989) but need updated confirmation |
| 18 | Bugim – Susana – São Domingos | NA | NA | Forest, agriculture, mangroves, roads, villages | King colobus  Temminck’s red colobus | Reported by Gippoliti and Dell’Omo (2003)  Unconfirmed |
| 19 | (31) Rio Cacheu Natural Park/Pelundo Faunal Reserve | 886 | NR | Mangroves, forest, open forest, woodland | King colobus  Temminck’s red colobus | Both species unsighted by Limoges (1989) but need updated confirmation |
| 20 | Cantchungu, Quinhàmel and Bissorã | NA | NA | Coastal forests (fragmented), mangroves, roads, villages, agriculture | King colobus  Temminck’s red colobus | Unknown  Unknown |
| 21 | Mansoã Forest Reserve | 91 | NR | Woodland, riverine forest, roads, agriculture, villages | King colobus  Temminck’s red colobus | Both species unsighted by Limoges (1989) but need updated confirmation |
| 22 | (32) Dungal Forest Reserve | 66 | NR | Open forest, woodland, roads, village, agriculture | Temminck’s red colobus | Reported by Limoges (1989) but needs updated confirmation |
|  | Guinea, Boké |  |  |  |  |  |
| 23 | (33) Iles Tristao Ramsar Site | 850 | NR | Coastal forests (fragmented), mangroves, woodland, grassland, agriculture, villages, roads | King colobus  Temminck’s red colobus  Chimpanzee | Unknown  Unknown  Reported by Ham (1997) |
| 24 | Kandiafara – along the river Kogon/border with Guinea-Bissau | NA | NA | Riverine forest, woodland, grassland, villages, agriculture | King colobus  Temminck’s red colobus  Chimpanzee | Unknown  Unknown  Unconfirmed |
| 25 | Kanfarandé | NA | NA | Coastal forests (fragmented), mangroves, open forest, agriculture, villages, roads | King colobus  Temminck’s red colobus  Chimpanzee | Unknown  Unknown  Yes (Leciak et al., 2005), but unknown distributions |
| 26 | (34) Badiar National Park | 382 | II | Woodland, grassland, open forest, riverine forest | Chimpanzee | Unknown |
| 27 | Guélémangaya area including coastal zones | NA | NA | Riverine forest, coastal forests (fragmented), open forest, agriculture, villages, road | King colobus  Chimpanzee | Unknown  Reported east of Guélémangaya by Ham (1997) |
|  | Tri-border area |  |  |  |  |  |
| 28 | Canquelifá, northeastern Guinea-Bissau, Missira, northwestern Guinea and Mampaye, southeastern Senegal | NA | NA | Open forest, woodland, riverine forest, villages, agriculture, roads, dam | Temminck’s red colobus  Chimpanzee | Reported present in Canquelifá, Guinea-Bissau by Limoges (1989)  Unknown |

^1^Galat et al. (2009) conducted surveys in 1975–2002 across southern Senegal but do not specify if they surveyed the particular location reported in this table. We therefore report it here as presence ‘Unknown’.
