# Supplementary material for: Primate occurrence across a human-impacted landscape in Guinea-Bissau and neighbouring regions in West Africa: using a systematic literature review to highlight the next conservation steps
Source: PeerJ. 2018 May 23;6:e4847. doi: 10.7717/peerj.4847 (PMC5970555; doi:10.7717/peerj.4847)
Supplement: Supplemental Information 4 [file peerj-06-4847-s004.docx]

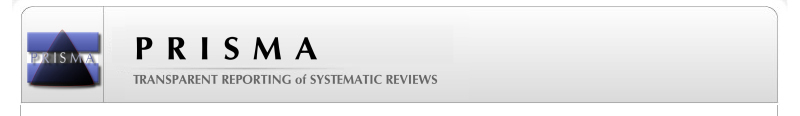
**PRISMA 2009 Flow Diagram**

Studies included in quantitative synthesis (meta-analysis)
(n = NA)

Studies included in qualitative synthesis
(n =151)

Full-text articles excluded, with reasons
(n =0)

Full-text articles assessed for eligibility
(n =151)

Records excluded
(n = 270)

Records screened
(n = 421)

Records after duplicates removed
(n = 421)

Additional records identified through other sources
(n =30)

Identification

Eligibility

Included

Screening

Records identified through database searching
(n =391 (Web of Science))
